# Periarticular Osteophytes as an Appendicular Joint Stress Marker (JSM): Analysis in a Contemporary Japanese Skeletal Collection

**DOI:** 10.1371/journal.pone.0057049

**Published:** 2013-02-20

**Authors:** Toshiyuki Tsurumoto, Kazunobu Saiki, Keishi Okamoto, Takeshi Imamura, Junichiro Maeda, Yoshitaka Manabe, Tetsuaki Wakebe

**Affiliations:** 1 Department of Macroscopic Anatomy, Graduate School of Biomedical Science, Nagasaki University, Nagasaki, Japan; 2 Department of Orthopaedic surgery, Graduate School of Biomedical Science, Nagasaki University, Nagasaki, Japan; 3 Department of Oral Anatomy and Dental Anthropology, Graduate School of Biomedical Science, Nagasaki University, Nagasaki, Japan; Museo Nazionale Preistorico Etnografico ‘L. Pigorini’, Italy

## Abstract

**Objective:**

The aim of this study was to investigate the possibility that periarticular osteophytes plays a role as a appendicular joint stress marker (JSM) which reflects the biomechanical stresses on individuals and populations.

**Methods:**

A total of 366 contemporary Japanese skeletons (231 males, 135 females) were examined closely to evaluate the periarticular osteophytes of six major joints, the shoulder, elbow, wrist, hip, knee, and ankle and osteophyte scores (OS) were determined using an original grading system. These scores were aggregated and analyzed statistically from some viewpoints.

**Results:**

All of the OS for the respective joints were correlated logarithmically with the age-at-death of the individuals. For 70 individuals, in whom both sides of all six joints were evaluated without missing values, the age-standardized OS were calculated. A right side dominancy was recognized in the joints of the upper extremities, shoulder and wrist joints, and the bilateral correlations were large in the three joints on the lower extremity. For the shoulder joint and the hip joint, it was inferred by some distinctions that systemic factors were relatively large. All of these six joints could be assorted by the extent of systemic and local factors on osteophytes formation. Moreover, when the age-standardized OS of all the joints was summed up, some individuals had significantly high total scores, and others had significantly low total scores; namely, all of the individuals varied greatly in their systemic predisposition for osteophytes formation.

**Conclusions:**

This study demonstrated the significance of periarticular osteophytes; the evaluating system for OS could be used to detect differences among joints and individuals. Periarticular osteophytes could be applied as an appendicular joint stress marker (JSM); by applying OS evaluating system for skeletal populations, intra-skeletal and inter-skeletal variations in biomechanical stresses throughout the lives could be clarified.

## Introduction

Marginal osteophytes are small protrusions of bone that may develop around the periphery of joints. These osteophytes emerge and grow gradually throughout a lifetime with the ageing process from physiological reactions to pathological conditions, in what is called degenerative joint disease (DJD). Analyzing the distributions of these alterations throughout the skeletal system can provide insights into a disturbed homeostasis that occurs in joints with DJD [1], but also these periarticular marginal osteophytes in vertebrate articulations can be probable and useful resources for studies on bioarchaeology.

Originally, synovial cartilage on bearing surfaces of most joints is not very strong, and, therefore, the loads being transferred from one bone to the next must be spread out over a rather large area of cartilage. Many vertebrate joints have a large angle of excursion, and the requirement for low stress implies large radii of curvature [2]. Osteophytes formation seems to be associated with these issues and progresses during physical ageing and pathological conditions such as osteoarthritis. Thus, periarticular osteophyte formation itself is a proliferative phenomenon. Moreover, it is one of the most discriminative changes in articular degeneration. Therefore, certainly they reflect the continuous biomechanical stresses placed on appendicular joints throughout one's life.

Periarticular osteophytes around all of the appendicular joints are themselves age-related phenomena. Thus, we thought that they should be evaluated appropriately using an age-standardized osteophyte scoring system. With such an original scoring system, we investigated periarticular marginal osteophytes of six major joints in a sizable skeletal population whose ages-at-death had been exactly recorded. The aim of this study was to investigate whether periarticular osteophytes play a role as an appendicular joint stress marker (JSM) which reflects the biomechanical stresses on individuals and populations. Intra-skeletal and inter-skeletal variations in osteophytes formation were evaluated to clarify the activity patterns and magnitudes among the individuals and the populations.

## Materials and Methods

### (1) Materials

A total of 366 modern Japanese skeletons (231 males, 135 females) were examined macroscopically. They were obtained from the cadavers that had been provided to Nagasaki University School of Medicine for anatomical dissection by medical students between the 1950s and 1970s, and most of them were voluntarily donated, and nowadays most of them were anonymous subjects.

After they had been dissected, their soft tissues were almost entirely removed from their bodies to produce dry skeletal preparations. Skeletons with some pathological conditions, for example, rheumatoid arthritis, infectious diseases, fractured joints, were excluded from this study. This was because the accidental influences by systemic diseases, metabolic diseases and injuries should be excluded in order to examine the effects of increasing age on the periarticular osteophyte formation. The sex and ages-at-death of all the individuals were registered exactly; the mean age-at-death was 64.4 years old (males: 61.8; females: 68.7 range: from 20 to 89) (Table 1).

**Table 1:** Age-at-death and sex distribution of the samples.

Age	male	female	total
20–29	8	2	10
30–39	11	3	14
40–49	31	12	43
50–59	39	7	46
60–69	63	28	91
70–79	57	54	111
80–89	22	29	51
Total	231	135	366

In each individual, six major joints, the shoulder, elbow, wrist, hip, knee, and ankle, were visually examined. The evaluated joint components were as follows; i) the humeral head and glenoid fossa for the shoulder joint, ii) the distal humerus, proximal ulna, and proximal radius for the elbow joint, iii) the distal radius and distal ulna for the wrist joint, iv) the acetabulum and femoral head for the hip joint, v) the distal femur, patella, and proximal tibia for the knee joint, and vi) the distal tibia, distal fibula, and the upper surface of talus for the ankle joint.

### (2) Osteophyte scoring system

**Figure 1:**
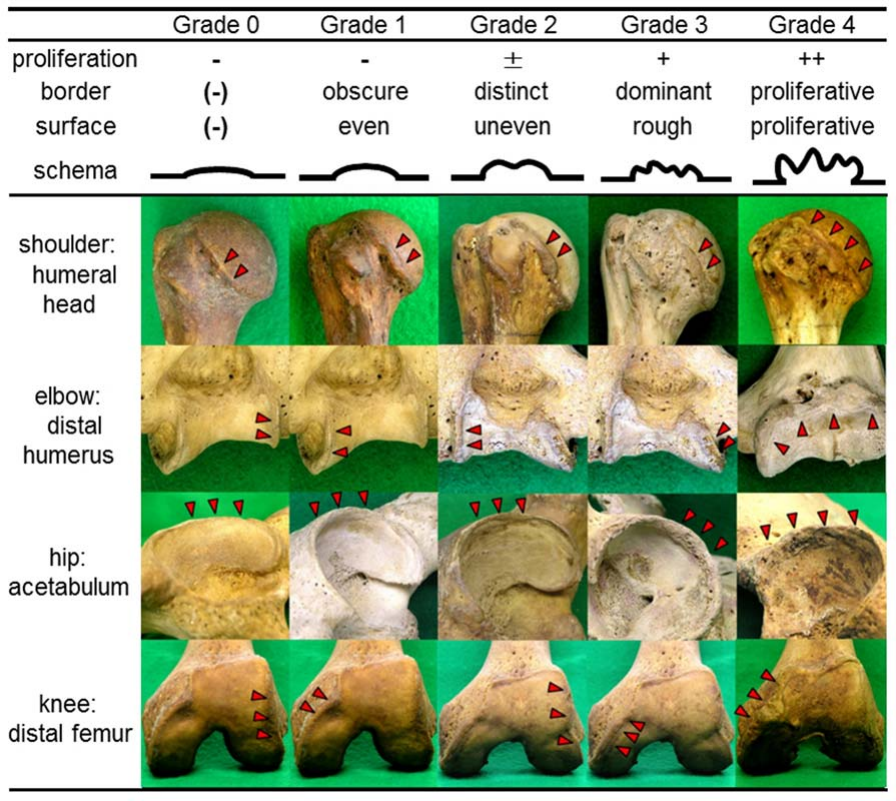
Periarticular marginal osteophytes: criteria for Grade 0, 1, 2, 3, and 4. Schemas and typical four articular components are shown (humeral head in shoulder joint, distal end of humerus in elbow joint, acetabulum in hip joint, and distal femur in knee joint).

The osteophytes in marginal regions were visually examined and graded 0, 1, 2, 3, or 4 according to criteria assessing osteophyte proliferation and the appearance of the border and surface of the joint (Figure 1). Marginal regions without any eminences were regarded as grade 0. Marginal osteophytes with an obscure border and even surface were graded 1. Marginal osteophytes with a distinct border and uneven surface were graded 2. Marginal osteophytes with a dominant border and rough surface were categorized as grade 3. Marginal osteophytes that displayed severe proliferation both at their border and on their surface were classified as grade 4. To improve the objectivity and stability of the osteophyte scoring system, more than fifty arbitrarily chosen skeletons were examined in a preliminary study, and then the system was reviewed. Finally, all of the skeletons were consecutively evaluated by one of the authors (TT).

### (3) Stepwise determination of the osteophyte score (OS)

**Figure 2:**
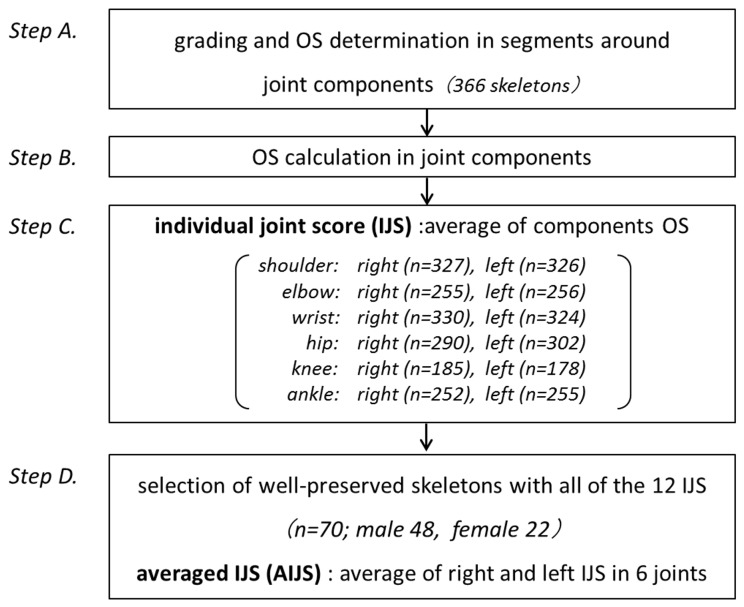
Flow chart describing our method for the steps in determining the osteophyte scores (OS): Step A: The OS for each segment was determined from its osteophyte grades. Step B: The OS for each joint component was determined by averaging all of its segmental scores. Step C: Individual joint scores (IJS) were determined by averaging the scores for all of its components. Step D: Well-preserved 70 individuals were selected, and for them averaged individual joint scores (AIJS) were determined by averaging the IJS of the right and left sides.

The marginal regions of each joint component were divided into an appropriate number of segments; for example, the humeral proximal joint margin was divided into eight segments, and the glenoid margin was divided into four segments. The OS for each segment was determined from its osteophyte grades; i.e., grade 0, 1, 2, 3, and 4 resulted in OS of 0, 10, 20, 30, and 40, respectively (*step A* in Figure 2). When a segment contained areas with various grades, its OS was determined by averaging the score for each area within the segment. The OS for each joint component was determined by averaging all of the segmental OS (*step B*). Incomplete joint components; i.e., those for which less than half of their segments were examined, were excluded from the analysis. The OS for all components in a joint were averaged to determine the individual joint score (IJS) (*step C*); for example, the score for the shoulder joint was calculated by averaging the OS for the humeral head and the glenoid fossa of the scapula. The joints missing one or more joint components were excluded from the analysis. Accordingly, 70 individuals (48 males, 22 females) with all of the twelve IJS (six joints on the right and left sides) were selected (*step D*); for them, the averaged IJS (AIJS) was calculated by averaging the IJS for the right and left sides.

### (4) Analysis of the individual joint score (IJS) obtained in *step C*

The right and left IJS values of all six joints were analyzed as follows; (i) Comparison of the right and left IJS: For all six joints, the right and left IJS of the both sexes were compared using the paired t-test with p<0.05 and p<0.01 (two-tailed). (ii) Analysis of the mean IJS for each joint according to decennial age group; For each of the joints, the mean IJS of decennial age groups ranging from 20–29 years old to 80–89 years old were calculated and plotted on line charts. The significance of the differences between the right and left sides was tested using the paired t-test with p<0.05 and p<0.01 (two-tailed), and the significance of the differences between the values for males and females was tested using the Student's t-test with p<0.05 and p<0.01 (two-tailed).

### (5) Analyses of joint scores obtained in *steps D*

(i) Correlation analysis on IJS; For all of the well-preserved 70 individuals, correlation coefficients in respective IJS values of both right and left sides in six joints were analyzed in a cross table with p<0.05 and p<0.01. (ii) Calculation of age-standardized IJS values; For all of the 70 individuals examined in step D, “age-standardized IJS” values that took the age of each individual into account were calculated with non-linear equations; these values were equal to the residual errors between the IJS and the prediction values in non-linear regression analysis. (iii) Analysis of 70 individuals with the sum values of age-standardized IJS; For all of the 70 individuals, the sum values of age-standardized IJS were calculated, and these individuals were arranged on a table in the order of these values to distinguish the individuals with extremely high total scores and those with extremely low total scores. (iv) Principal component analysis (PCA) and cluster analysis in 70 individuals; The characteristics in the respective joints were evaluated with principal components analysis (PCA); a variety of the combinations of age-standardized AIJS values in six joints of the 70 individuals were evaluated with the statistical software JMP (version 10.0.2) (SAS Institute Inc.). Moreover, cluster analysis using the values of age-standardized IJS in 12 joints of the 70 individuals was applied to assess how individual joints grouped with respect to each other. The open source programming language “R” (version 2.15.1) was used for characterizing cluster dendrogram, and its reliability was assessed with the bootstrap probability (BP) values.

## Results

### (1) Comparison of the right and left IJS values (table 2)

**Table 2:** Average values of individual joint scores (IJS) for the right and left sides of the 12 appendicular joints (*p<0.05, **p<0.01).

		IJS ± S.D.		
		right		left
Shoulder	male	24.9±9.0	>^*^	23.9±8.7
	female	21.6±9.2	>^**^	19.5±7.0
Elbow	male	20.6±8.9		21.0±10.4
	female	20.6±8.7		20.0±8.5
Wrist	male	21.3±8.2		21.2±8.0
	female	18.7±6.5	>^**^	17.6±6.1
Hip	male	28.9±6.1		29.0±6.3
	female	27.6±5.7		27.7±5.6
Knee	male	18.5±7.6		19.5±7.8
	female	19.7±9.0		18.8±8.8
Ankle	male	11.0±5.1		10.5±4.7
	female	13.0±4.7		13.6±5.8

Both sides of the shoulder joint and hip joint showed relative high IJS values. Some joints in the upper extremities showed the dominancy of the right side; the IJS for the right shoulder in both sexes and those for the right wrist in females were significantly higher than those for the same joints on the left side with the paired t- test. Most people from different geographic regions are right handed; therefore, high IJS values of the right sides in the shoulder and wrist joints were reasonable.

### (2) Analysis of the mean IJS values for each joint among decennial groups (Figure 3)

The mean IJS of each joint were analyzed among decennial groups as follows. (i) Shoulder joint: In the 60 to 79-year-old females, the mean IJS values for the right side were higher than those for the left side. The mean IJS values of the males continued to increase steadily until extreme old age and were higher than those of the females in the over sixties age group. (ii) Elbow joint: There were few differences between the right and left sides or between the sexes. (iii) Wrist joint: There were few differences between the mean IJS values of the right and left sides, but in males the mean IJS for both sides were significantly higher than those of the females in the 60 to 79-year-old age group. The distribution of the mean IJS for the wrist joint resembled that of the shoulder joint. These occasional high scores of the right-side upper extremities inferred the right side dominance in most individuals. (iv) Hip joint: The mean IJS values in the males seldom increased after middle age, and those of the females increased gradually. The former were consistently higher than the latter in all age groups. (v) Knee joint: All of the males displayed similar mean IJS after forty years of age, but those of the females continued to increase steadily until extreme old age and overtook the males in the older groups. (vi) Ankle joint: The males displayed similar averages after forty years of age, but those of the females continued to increase steadily until extreme old age. The values for the females were higher than those of the males in the over 70-year-old age group. The distribution of the mean IJS for the ankle joint resembled that of the knee joint.

### (3) Calculation of age-standardized IJS values

**Figure 3:**
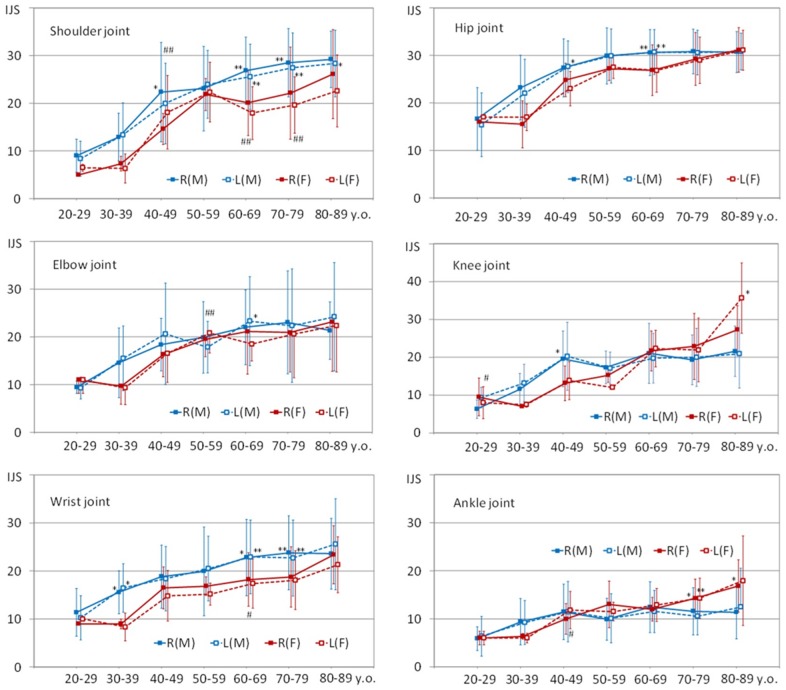
Individual joint scores (IJS) for the shoulder, elbow, wrist, hip, knee, and ankle joints of decennial age groups (mean and standard deviation (bar line) values). R(M) blue full line; males, right L(M) blue dotted-line: males, left. R(F) red full line; females, right L(F) red dotted-line; females, left. Significant difference between right and left sides (# p<0.05, ## p<0.01). Significant difference between males and females (* p<0.05, ** p<0.01).


Considering the result of analysis about the mean IJS values for each joint among decennial groups (Figure 3), the relationships between the IJS and the chronological ages of the individuals approximated with logarithmic non-linear correlations. Natural logarithmic functions in respective joints of the males and females were determined by analyzing the non-linear regression between the chronological ages and the IJS values of each joint (n = 70);

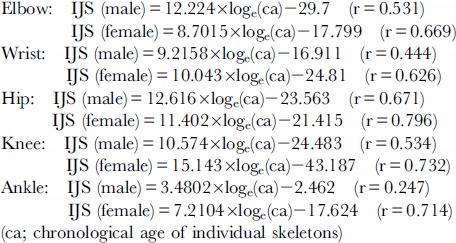


**Figure 4:**
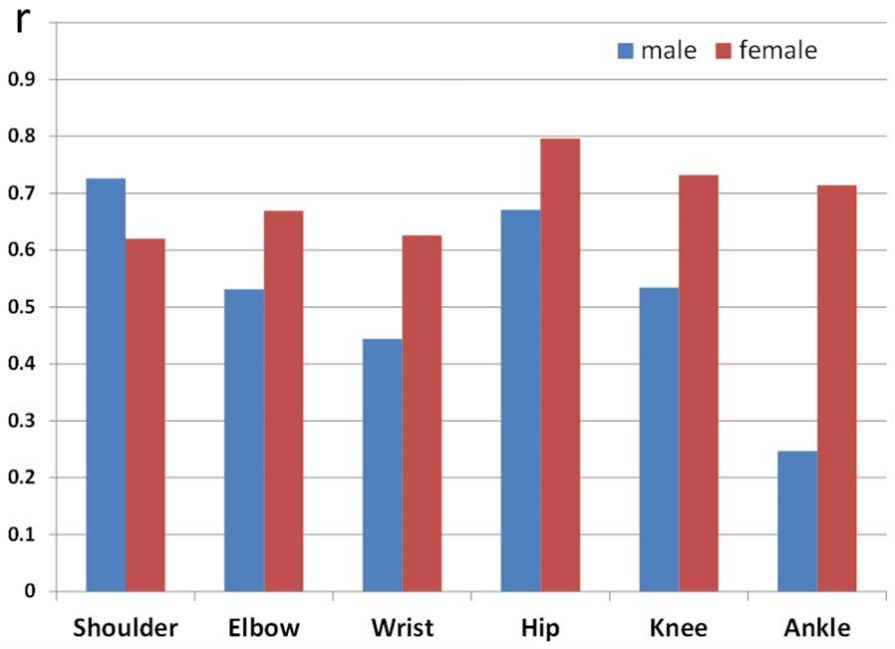
Values of correlation coefficients in non-linear, logarithmic regression analysis between IJS and chronological age (blue: male, red: female).

These correlation coefficients in non-linear regression analysis between IJS and chronological age are shown in Figure 4. Regarding these values, five joints (other than the shoulder) showed the dominancy of the female. The shoulder and hip joints, which are located at the proximal positions in the upper and lower extremities, had high values more than 0.7; moreover, the distal joints had lower coefficients. In particular, the ankle joint in the males showed the lowest coefficient, 0.247. On the other hand, all of the female joints had values higher than 0.6; especially the coefficients of all three joints in the lower extremity were as high as 0.7.

### (4) Correlation analysis of IJS in 70 individuals

**Figure 5:**
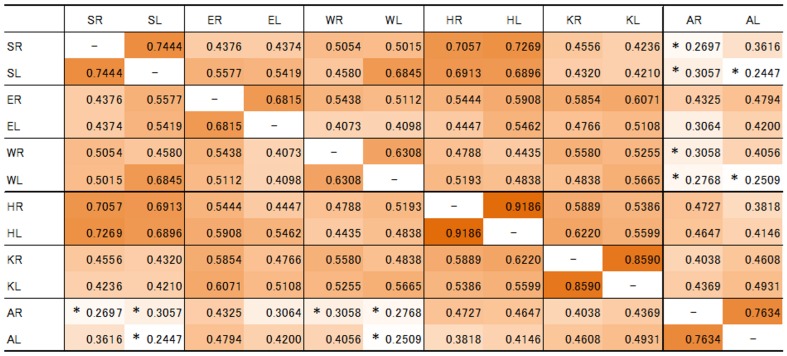
Correlation coefficients between AIJS values in respective joints among the well-preserved 70 individuals; the high values are shown in dark brown and lower values were in light brown or white (* p<0.05, no mark p<0.01).

For the well-preserved 70 individuals, the correlation coefficients between AIJS values in respective joints are shown in figure 5. The high values are shown in dark brown boxes and lower values in light brown or white boxes in this cross table. Almost all of the joints (other than the ankle joints) had significantly high correlation coefficients. For all six major joints, the correlation coefficients between the right and left of the respective joints were high. Especially three joints of the lower extremities had high correlations; hip 0.9186, knee 0.8590, ankle 0.7634. Furthermore, between the shoulder joints and the hip joints, between the left shoulder and the left wrist joint, the correlation coefficients were relatively high. On the other hand, the ankle joints showed a low correlation with the others. Furthermore, between the shoulder and knee joints, between the shoulder and ankle joints, between the wrist and ankle joints, the correlation coefficients were rather low.

### (5) Analysis of 70 individuals with the sum total values of age-standardized IJS

**Figure 6:**
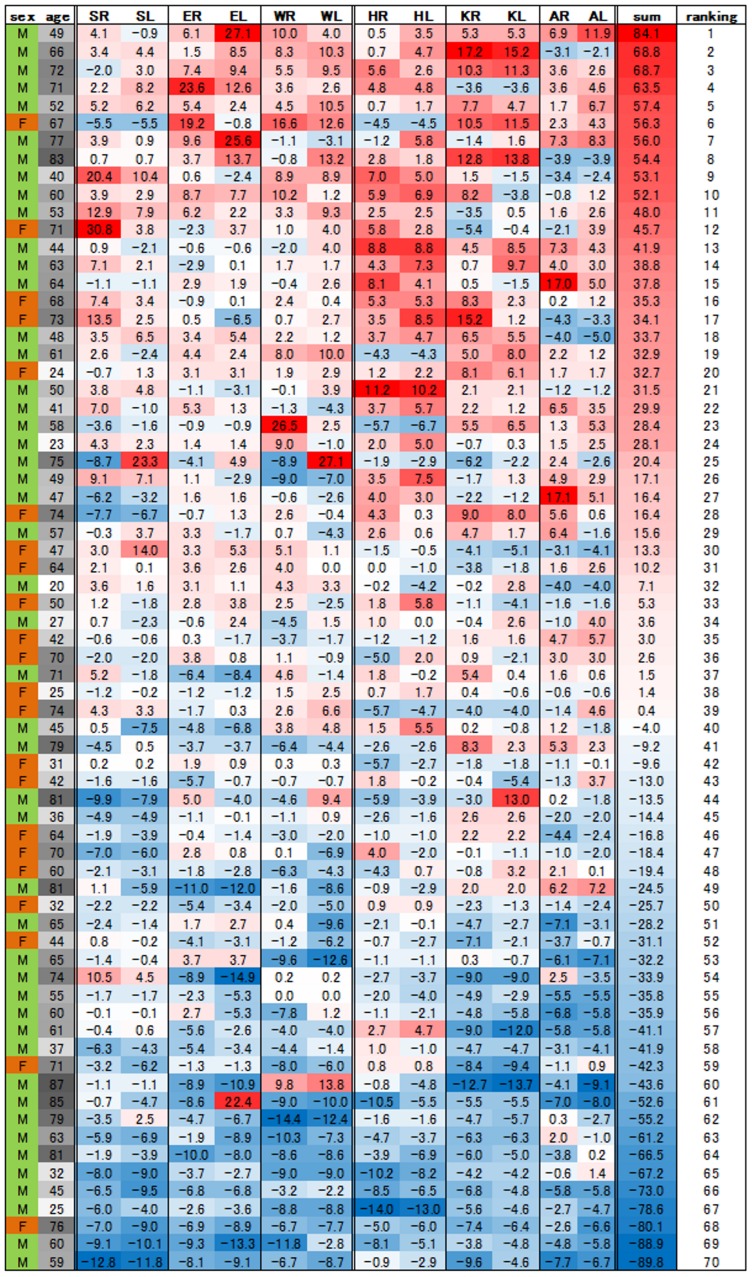
Tabulated list of age-standardized IJS in the well-preserved 70 individuals. Individuals are arranged in the order corresponding to the sum values of all of the 12 IJS; higher values in each column are shown in red and lower values in blue. The rightmost column indicated the ranking numbers from the highest as 1 to the lowest individuals as 70. (SR: right shoulder, SL: left shoulder, RE: right elbow, LE: left elbow, RW: right wrist, LW left wrist, RH: right hip, RL left hip, RK: right knee, LK: left knee, RA: right ankle and LA: left ankle).

**Figure 7:**
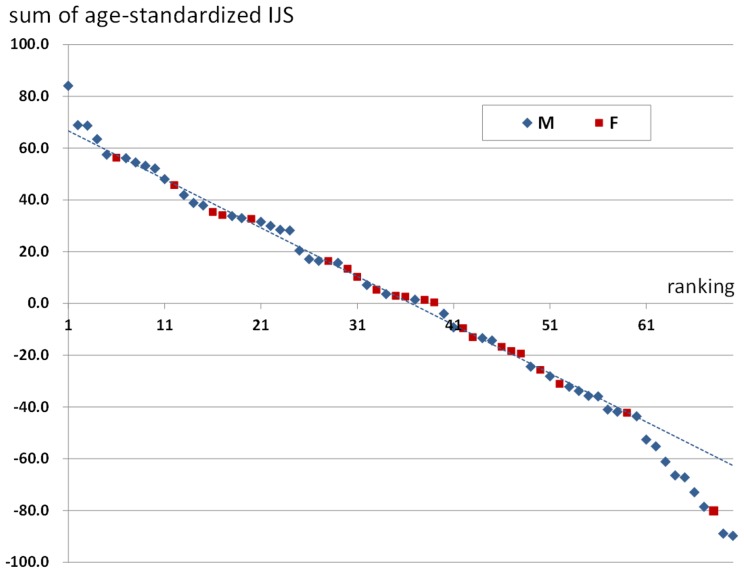
Scatter chart indicating the relationship between the sum of age-standardized IJS values of the 12 joints and the ranking numbers in the well-preserved 70 individuals (blue: male, red: female).

Figure 6 shows all of the age-standardized IJS in the 70 individuals; the high values in each column are shown in red and lower values in blue. The second column from the right listed the sum total values of each individual, and the rightmost column indicates the ranking numbers from the highest to the lowest individuals. There was a wide variability in the sum total values of respective individuals from the highest 84.1 to the lowest −89.8; this result indicated the difference among individuals in periarticular osteophytes formation. Moreover, some individuals had systemically almost equivalent values, and other individuals had uneven distributions. Figure 7 is the scatter chart which indicated the relationship between the sum of age-standardized IJS values of 12 joints and the ranking numbers. The great majority of the individuals showed a linear relationship, but the some individuals with high scores swerved from the line to higher values and, some individuals with low values, whose ranking number was 61 or more, dropped from the line to lower values.

### (6) Principal component analysis (PCA) and cluster analysis in 70 individuals

**Figure 8:**
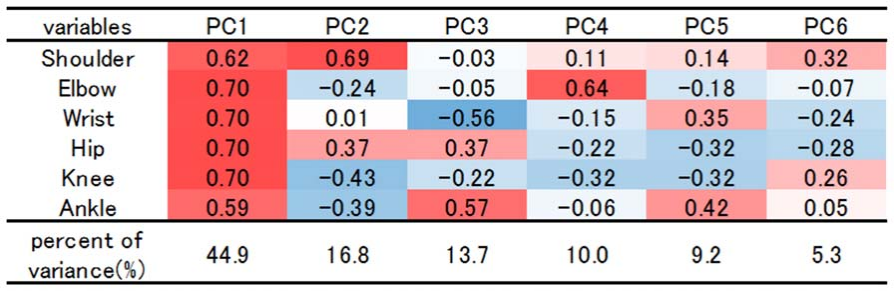
PCA variable loadings evaluating the characteristics in the respective six joints with age-standardized AIJS values; the high values are shown in red and lower values in blue.

Figure 8 indicates PCA variable loadings; the high values are shown in red boxes and lower values in blue. The first three PCs explained 65.4% of the total variance among taxa, with 44.9% on PC1, 16.8% on PC2, 13.7% on PC3. Loading values in all of the six joints were positive and more than 0.5 in PC 1; this axis was indicated to show the overall tendency of osteophyte formation in individuals. This meant that one of the most influential factors for osteophyte formation was the individual specificity. In PC2, the loading values of the shoulder joint and hip joint were high; both of them take proximal position in the extremities. On the other hand, loadings in the elbow, knee and ankle joints were low. In PC3 and PC4, joints in the upper extremity and joints in the lower extremity were almost decoupled. In PC5, joints in the peripheral position, wrist and ankle showed relatively high values.

**Figure 9:**
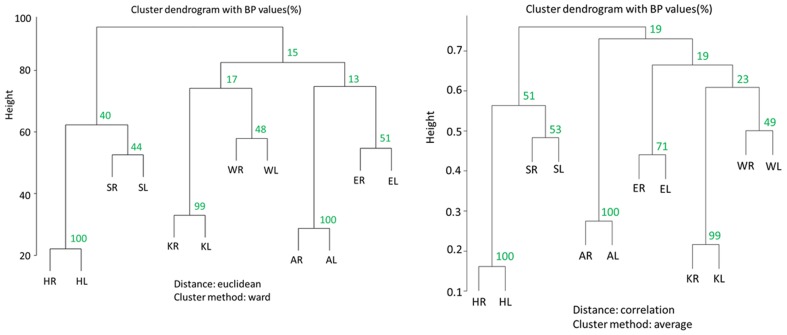
Results of cluster analysis showing the similarities in the 12 joints among the well-preserved 70 individuals (left; dendrogram chart with ward method, right; dendrogram with group average method).

Cluster dendrograms in Figure 9 showed several similarities in 12 joints among the well-preserved 70 individuals; Figure 9 shows the dendrogram charts analyzed with ward method (left) and with the group average method (right). Firstly, both dendrogram charts generally confirmed great similarity between right and left sides in respective joints. Especially, in the lower extremity, all of the three joints showed high BP values of 99 or 100%, and extremely close relationships with each opposite side were indicated. Compared with these joints in the lower extremity, both sides of the shoulder, elbow and wrist joints have relatively low BP values from 44 to 71%. Secondly, both dendrograms indicated the unexpected similarity between shoulder joints and hip joints. This similarity was confirmed by the axis 2 of PCA in Figure 8.

## Discussion

Osteoarthritis, or DJD, is a gradually progressing degeneration of appendicular joints. Basically, this is a complicated pathological condition which combines proliferative changes of osteophyte formation and often slight inflammations. The osteophyte formation closely resembles the processes of chondrogenesis and endochondral bone formation, which occur during embryogenesis [1]. Periarticular osteophytes emerge through the endochondral ossification of fibrous cartilage at the synovial bone-cartilage junction. In a number of disease conditions, the formation of abnormal calcifications and bony spurs seems to be related to changes in the composition of fibrocartilage in the attachment zone [3].

Both systemic factors and local factors have effects on DJD progression. The former contains age, sex, metabolic factors, nutrition, vascular profusion, endocrine factors and heredity, and the latter contains severe trauma and chronic functional stress [4] [5]. General obesity is one of the local factors, because it has a great effect on local mechanical stress to the lower extremities. Moreover, osteoporosis is a potent systemic factor, and the degree of inflammation and degeneration itself, which yields debris within articular space, have local effects on these formations. Moreover, some genetic aspects of osteoarthritis have been referred to; for example, the manifestation of generalized OA was shown to be dominant in females and recessive in males [6]. OA is a polygenic disease indeed, and some osteoarthritis susceptibility genes have been reported by many researchers [7] [8]. It is possible that these genetic factors work in concert to affect OA onset and progression.

For the purpose of clarification of these DJD developments and features, in this study, the six major appendicular joints of dried skeletons were observed and only the periarticular osteophytes were evaluated. They were strictly investigated by a simple and detailed evaluation system. There have been many studies which dealt with degenerative and proliferative changes of the appendicular joints. Some early studies dealt with the cadaver materials [9]. Then, Jurmain studied a total of 444 skeletons whose ages and sexes were relatively precisely known, called the Terry collection; he evaluated DJD in their four major joints by scoring marginal osteophyte and articular surface conditions in some parts of the joints, and this yielded much useful information on DJD [10]. Like Jurmain, some other studies adopted the direct observation techniques for skeletons. Moreover, there have been a lot of studies which evaluated the features in DJD by means of roentgenographic evaluation techniques [11]–[18], which were proposed originally by Kellgren and Lawrence [19]. X-ray goes through the objects to form two-dimensional images on the films; therefore, concerning the periarticular osteophytes, the evaluated area is only a narrowly restricted tangential portion of the joint margin [20] [21].

In this study, periarticular osteophytes were evaluated with our original scoring system. With this system, osteophytes were scored in many small segments in order to calculate a whole joint score. For example, one shoulder joint was divided to a total of 12 segments, and one knee joint was divided to a total of 19 segments. Jurmain once emphasized the significance of a fine grading system while overweighting the importance of osteophyte development to sense the DJD frequencies within different joints. Though quantification of these periarticular osteophytes was relatively easy, it was difficult to evaluate the articular surface condition with unitary criteria; this was because a characteristic degenerative change in one joint might not be observed in another [10]. Merely for the purpose of studying the frequency of DJD in the skeletons, it might be better to evaluate the highest scores in respective joints. However, the purpose of this study was not only the evaluation of DJD frequency in a skeletal population but also the quantification of biomechanical stresses with the evidence in articular regions. Therefore, osteophytes located in periarticular regions were graded from 0 to 4, and the calculated scores were averaged for further evaluation. All of the appendicular joints were evaluated with common simple criteria, which made it possible to compare the different kinds of joints objectively. As described above, the periarticular osteophytes themselves are proliferative changes, and they often emerge as articular degeneration with ageing; they can be good biomechanical stress markers.

A good deal of study presented in the literatures, which discussed DJD prevalence on skeletal series from archaeological contest, were troubled by the fact that the most reliable age-at-death skeletal indicators (such as the changes of the sternal end of the ribs, the auricular surface and the pubic symphysis of the hip bone) were also related to the biomechanical stress during the individual's life [22]–[29]. On the other hand, in this study, as with Jurmain [10] [30] and Waldron et al. [31] [32], the skeletal individuals with exact records on sex and age-at-death were targeted, and this made it possible to discuss the features around osteophyte formation and ageing.

By considering the results of age correlation, bilateral correlation and the difference between the right and left sides in this study, the extent to which systemic or local factors got involved in the respective joints was clarified. From the result of the paired-T test, right side dominancy was recognized in the joints of the upper extremities, shoulder and wrist joint. This suggested that upper extremities were generally under the influence of local stress of various sizes. However, in the shoulder joint, the high bilateral correlation indicated the disparities among the individuals; so not only the local stress but also the general osteophyte proliferation tendency might be high in this joint.

The correlation coefficients between the right and left side of the lower extremities were high, especially in females. This could be explained by the fact that the female's scores continued to increase after middle age. One of the reasons might be the high morbidity rate of osteoarthritis in the knee joints of middle-aged and older females in Japan. Conversely, in males, age correlation of scores in knee and ankle joints was low. This meant that the local stresses, for example with trauma or injuries, were high in these male joints. These sex discrepancies in IJS were considered to reflect the differences in lifestyle, habitual activities, body habitus, metabolic/endocrinal condition and the receptivity to DJD among Japanese males and females. The individuals in this study lived their lives between the last decades of nineteenth century and mid-twentieth century, and most of them survived the extreme and hard days before and after World War II. Though the samples in this study were too heterogeneous to elaborate on these differences, all that was certain is that most Japanese women from these generations habitually sat down “Japanese style” with their buttocks on top of the ankles; it must lead to the high prevalence of DJD in the lower extremities.

From the viewpoint of periarticular osteophyte formation, there was a similarity in the shoulder joint and the hip joint; it was inferred that systemic factors were indicated to be relatively large in these two joints. This feature was consistent with the results on both PC2 loading values in PCA and dendrogram in cluster analysis. These two joints are relatively mobile from the biomechanical homologies in that the components are almost spherical, and in that both of them are multiaxial. Moreover, they are similarly located at the proximal positions in the upper and lower extremities. These anatomical and biomechanical analogies might explain the similarly steady progressive pattern in osteophyte formation by ageing. Jurmain reported that the age correlations of the shoulder and hip joints were higher than those of elbow and knee joints in the Terry collection and that the bilateral correlation in the elbow joint was low [10]. By quoting Fischer's [33] observation, Jurmain referred to the asymmetry of elbow joints and great stress in this joint [10]. The results in this study mostly confirmed their finding.

Radiographic changes of DJD, particularly osteophytes, are common in the aged population, but symptoms of joint pain may be independent of radiographic severity in many older adults [34]. Indeed, one of the impressive results in this study was the relative high osteophyte scores in shoulder and hip joints of males and females. Clinically the frequency of DJD of the knee is highest among the appendicular joints in Japanese people; on the other hand, generally the hip joint is seldom affected with primary osteoarthritis. Disjunction was noted between these facts and the results of this study; therefore, osteophyte formation must be one of the distinct phenomena in joints with DJD, and it was doubted that all of the formed osteophytes were not related to DJD. In this study, osteophytes scores in almost all of the joints increased logarithmically with ageing. Namely, it was speculated that periarticular osteophytes started to grow rapidly before the symptoms of DJD became apparent. Though most of the joints stopped showing increasing osteophyte scores after middle age, some of the joints in the male's upper extremity and in female's lower extremity continued to increase even in the elderly. In these joints osteophytes formation must be related to DJD progression. Indeed, in Japan, one of the dominant joints with DJD is females' knee joint, though it would be impossible to explain the difference of physical activities. Hernborg et al. confirmed that osteophytes were frequently observed in cases that later on developed osteoarthritis and continue to grow in size in these cases at a faster rate than in cases that did not develop osteoarthritis with structural changes in the joint [35]. On the other hand, there were osteophytes which stopped growing after middle age; they might not be pathological but physiological.

Chronological ages of the individuals are thought to have a profound effect on the degree of periarticular osteophytes. In this study, the age-standardized osteophyte scores were investigated among the individuals; this made it possible to compare the osteophyte proliferation size and grade in different kinds of joints. As indicated in Figure 7, osteophytes proliferation tendency in a whole body showed wide individual variation. Especially on both sides of the scatter chart, there existed some individuals with extremely high or low scores. Rogers et al. pointed out that osteophyte and enthesophyte proliferations are linked; thus, individuals in populations can be classified into “bone formers” and “poor bone formers” [36] [37]. Some factors should be pointed out as backgrounds of these individual differences; among the various factors listed above, the systemic factors for DJD progression can have effects on the differences in bone formation. For example, genetic factors might significantly contribute to these tendencies.

Moreover, recently it has been reported that some kinds of calcifying nanoparticles might make ectopic calcifications and trigger some pathological conditions in many organs in human bodies, for example, heart valves [38] [39], urinary bladder [40], prostate [41], placenta [42], and synovial joints [43]. If these nanoparticles increased in the synovial joint spaces, ectopic calcification, ossification and osteophyte formation could be accelerated around the joint surfaces. Moreover, Timms et al. confirmed in his review of the genetic potentials in the patients with chondrocalcinosis due to calcium pyrophosphate deposition (CPPD), diffuse idiopathic systemic hyperostosis (DISH) and ossification of posterior longitudinal ligament (OPLL) [44]; and the generalized osteoarthritis (GOA) should be considered in the same category [45]. Some genetic studies targeting the individuals with high or low osteophyte scores in this study might yield a meaningful result in the future.

It has been emphasized that the degenerative changes in peripheral joints could be used as the markers of mechanical stresses; and frequently they have been applied to reconstruct the past lifestyles and activity patterns and to clarify the differences among human groups [46]–[50]. This study suggests that only in cases in which age is standardized properly can periarticular osteophytes be used as a sensitive biomarker for indicating abnormal phenomena in the appendicular joints, osteoarthritis severity, and the biomechanical stresses placed on individuals and populations. Even if some individuals and some joints dominantly are affected by the influence of systemic factors, they cannot but undergo various local biomechanical stresses. Therefore, osteophytes could play a significant role as a “joint stress marker (JSM)” in a similar manner to “musculoskeletal stress markers (MSM)”, which is a term used for the sites of origin and insertion of muscle tendons (entheses) and ligament attachments (syndesmoses) in the skeleton [51]–[53]. Additional analyses targeting the marginal osteophytes of both joint components and of independent parts in joint surfaces would be useful for studying poorly preserved archeological samples. For these samples, some interesting and valuable findings might be able to be confirmed with some comparison/correlation analyses, PCA and cluster analysis.
